# A Novel Artificial Organic Control System for Mobile Robot Navigation in Assisted Living Using Vision- and Neural-Based Strategies

**DOI:** 10.1155/2018/4189150

**Published:** 2018-12-02

**Authors:** Hiram Ponce, Ernesto Moya-Albor, Jorge Brieva

**Affiliations:** Universidad Panamericana, Facultad de Ingeniería, Augusto Rodin 498, Ciudad de México 03920, Mexico

## Abstract

Robots in assisted living (RAL) are an alternative to support families and professional caregivers with a wide range of possibilities to take care of elderly people. Navigation of mobile robots is a challenging problem due to the uncertainty and dynamics of environments found in the context of places for elderly. To accomplish this goal, the navigation system tries to replicate such a complicated process inspired on the perception and judgment of human beings. In this work, we propose a novel nature-inspired control system for mobile RAL navigation using an artificial organic controller enhanced with vision-based strategies such as Hermite optical flow (OF) and convolutional neural networks (CNNs). Particularly, the Hermite OF is employed for obstacle motion detection while CNNs are occupied for obstacle distance estimation. We train the CNN using OF visual features guided by ultrasonic sensor-based measures in a 3D scenario. Our application is oriented to avoid mobile and fixed obstacles using a monocular camera in a simulated environment. For the experiments, we use the robot simulator V-REP, which is an integrated development environment into a distributed control architecture. Security and smoothness metrics as well as quantitative evaluation are computed and analyzed. Results showed that the proposed method works successfully in simulation conditions.

## 1. Introduction

Nowadays, there is a dramatic increase in the aging of the population. It is expected that the number of people over 60 years will go from 962 million in 2017 to 1.4 billion in 2030 and 2.1 billion in 2050 [1]. Along with this increasing in elderly people and consequently higher life expectancy, there is a necessity of the creation of new care strategies. This problem is aggravated by the shortage of professional caregivers and their high costs. Some experts argue that it is desirable for elderly to stay in their own home with a certain level of independence and a sense of comfort and security. However, it requires to maintain an acceptable quality of life and independence capability [2].

Robots in assisted living (RAL) are an alternative to support families and professional caregivers with a wide range of possibilities to take care of elderly people. There are many issues where robots have high potential for assistance such as social isolation, diminishing independent living, physical and cognitive impairment, loss of mobility, lack of recreation, and risk of falls. These problems can be tackled with different robot designs categorized as service, assistance, social, and rehabilitation robots [2].

Autonomous navigation is a challenging problem required in RAL due to the uncertainty and dynamics of the environments. A robot must be aware of where it is in relation to the surrounding environment and to localize itself during all the time. Hence, robot navigation skills must include different tasks as perception, exploration, mapping, localization, path planning, and path execution [3]. To accomplish this goal, the navigation system should replicate such complicated processes inspired on the perception and judgment of human beings. One approach to do so in robotic systems is the usage of vision sensors as fixed cameras located on the robot to process the video information for navigating in a given environment.

One of the challenges using vision sensors is the characterization of the 3D scene for computing features that can be used in the navigation system. Different approaches have been proposed to solve this complex problem like monocular- and stereovision-based systems. For instance, in stereo-based systems, depth can be computed directly with the known leaks in limitation of detection, camera setup, and low speed of the system [4]. In contrast, monocular systems can exploit the geometric restrictions of the scene with the inherent generalization problem [5], and they can use the apparent motion of objects in scene computed, for example, with methods based on optical flow (OF), in order to estimate depth in the scene.

In monocular applications, OF approaches have shown advantages regarding other methods specially in the relationship between spatial and temporal gradient [6]. These OF family methods are widely used in robotic applications assuming that the apparent velocity of the brightness pattern varies smoothly almost everywhere in an image [7]. It has been used for estimating depth of the scene [8, 9], relative motion [10], and apparent velocity estimation [11, 12].

Moreover, artificial intelligence (AI) has been widely used for navigation of robots in assisted living, obtaining different levels of cognition: reasoning, decision making, and learning. Recently, convolutional neural networks (CNNs) are used in a wide variety of computer vision and robotic applications such as depth and distance estimation [13–16]. In addition, obstacle detection using OF is presented in [17], and the authors in [18] developed distance estimation from robot to obstacles using CNN.

To get benefit from the combination of artificial intelligence and visual perception methods in RAL, this paper presents a novel nature-inspired control system for mobile robot navigation using an artificial organic controller enhanced with vision and neural-based strategies, i.e., Hermite OF and CNN using a monocular camera and tested in a simulated environment. Particularly, the Hermite OF is employed for obstacle motion detection while CNN is occupied for obstacle distance estimation. For the experiments, we use the robot simulator V-REP [19], which is an integrated development environment into a distributed control architecture. The simulated robot is equipped with only a monocular camera.

The contribution of this work is focused on the integration of raw OF features and the estimated object distance to the obstacle as input to the controller. The estimation of the object distance is performed using CNN. The latter is trained using the OF features as input and as reference for the distance measured using ultrasonic sensors. In addition, the whole set of features is used as input to the nature-inspired control system based on the artificial organic controller. We developed our approach from previous studies: a system employing a basic controller using only OF features [20] and the same optical features as input to an artificial organic controller [21]. All the experiments have been carried out using the same camera parameters.

The rest of the paper is arranged as follows: Section 2 describes the proposed approach, the experiments, and the protocol, and Section 3 discusses results and finally, conclusion is presented.

## 2. Materials and Methods

In this section, we present relevant studies associated to monocular depth estimation found in the literature. Then, it is explained the methods used in our approach and finally, the nature-inspired control system for mobile robot navigation is proposed.

### 2.1. Related Work

Monocular depth estimation can be tackled by CNN in two different ways: supervised and unsupervised. For instance, unsupervised methods include several approaches as discussed in [22], and the authors proposed a monocular depth estimation using a CNN trained without ground truth data. This proposal considered to exploit epipolar geometry constraints, giving as result a better depth map than traditional supervised learning methods. Another approach based on CNN and random forest is the proposal explained in [23]. The work presented a network trained by learning the parameters in an unsupervised way through maximizing the likelihood of the training data. In [24], a method using deep CNN to depth prediction without requiring a pretraining stage was reported. In [25], the authors proposed an unsupervised CNN-based method for explicit depth estimation from light field, which learns an end-to-end mapping from a 4D light field to the corresponding disparity map without the supervision of ground truth depth.

Several applications using supervised learning are presented in the literature. In [26], the authors described a depth estimation from monocular images using regression on deep features from a CNN and a conditional random field. The implementation considered two levels of depth inferring, pixel-to-pixel and regions of pixels. A similar approach was done in [27] but with discrete mapping inference. Gan et al. in [28] performed an explicit model to describe the relationships of images obtained from a monocular camera with an affinity layer and by combining absolute and relative features into a CNN, also local vertical features of depth estimation were incorporated. Cheng et al. [29] proposed a convolutional spatial propagation network (CSPN) to learn the affinity matrix for depth estimation from a single image. In [30], it is used a deep model to generate dense depth maps from a RGB image employing depth estimation of sparse set of pixels. In [31], deep structured model was presented in which the structured pixelwise depth estimation has ordinal constraints introduced by the user. In [32], the problem of estimating the depth map of a scene given in a single RGB image was solved by training a convolutional residual network to model the ambiguous mapping between monocular images and depth maps. However, most of the studies based on CNN suppose rigid scenes, as in the proposed method from [33]. In [34], depth map prediction employed two deep network stacks: the first makes a coarse global prediction on the whole image and in the second step the prediction is refined locally on the image.

Typically, monocular depth estimation considers that the captured scene is static and with constant depth. In practice, there is a blurring effect between camera and the objects. Thus, other methods such as deblurring and flow estimation are required, as shown in [35]. However, the approach reported the usage of stereo cameras. Other approaches for dynamic scenes are those based on motion estimation. In this case, OF between two consecutive images is also applied for depth estimation through motion segmentation, as proposed in [36]. In [37], a method to automatically estimate the depth of video frames of a single camera was proposed, and this estimation was carried out by analyzing the OF of preexisting videos and by using a pretrained CNN.

An example of robot navigation and localization using monocular depth estimation can be found in [38]. It showed that using CNN for depth estimation combining with monocular simultaneous localization and mapping (SLAM) can be successfully applied.

### 2.2. Optical Flow

OF is a 2D distribution of apparent velocities associated, usually, with intensity pattern variations in a sequence of images, and it is represented by a vector field that encodes the displacement for each pixel in the image sequence.

There are many approaches to obtain a dense and accurate OF estimation, where it is well known that the differential methods overcome other ones [39]. Those are based on the work of Horn and Schunck [7], which assume that the intensity of the objects remains constant during small periods of time and that the neighboring pixels have similar displacement. This method has low computational time, but it cannot handle large displacements. Recent OF approaches are more accurate to large displacements, but they are computationally expensive and very difficult to implement in devices with limited hardware.

In this paper, OF proposal is based on the studies of Moya-Albor et al. [20] and Ponce et al. [21]. It uses the Hermite transform [40], as bioinspired image model, to incorporate local descriptors allowing to describe the intensity and gradient constraints found in the current methods. This model increases the accuracy of the Horn and Shunck method, and it is more robust to noise.

### 2.3. Convolutional Neural Networks

CNN is a well-known neural network architecture inspired on the nature of visual perception in living creatures [41] typically applied for classification and regression in image processing [42]. There exists different architectures of CNN, but it is mainly constituted by three types of layers, namely, convolutional, pooling, and fully connected. The first layer aims to compute feature representations of the input, a pooling layer aims to reduce the resolution of feature maps, and a fully connected layer aims to perform high-level reasoning [41]. Lastly, a CNN may include an output layer aiming to compute the classification or regression task. Particularly, image and video applications have been widely explored with CNN.

### 2.4. Artificial Organic Controllers

An artificial organic controller (AOC) is an intelligent control strategy aiming to compute the control law using an ensemble method, namely, fuzzy-molecular inference (FMI) system [43]. It consists of a hybrid method from both fuzzy logic and artificial hydrocarbon networks (AHN). To properly design the AOC for the proposed robot system, an overview of AHN as well as the FMI system is introduced as follows.

#### 2.4.1. Overview of Artificial Hydrocarbon Networks

In machine learning, AHN algorithm is a supervised learning method inspired on the inner mechanisms and interactions of chemical hydrocarbon compounds [44]. This method aims to model data points like packages of information, called molecules. The interaction among these units allows capturing the nonlinearities of data correlation. From this point of view, an artificial hydrocarbon compound is built, and it can be seen as a net of molecules. If required, more than one artificial hydrocarbon compound can be added up to finally get a mixture of compounds [45].

In AHN, the basic unit of processing information is the molecule. It performs an output response *φ*(*x*) due to an input *x* ∈ ℝ^*k*^, as expressed in Equation (1) where *v*_C_ ∈ *ℝ* represents a carbon value, *h*_*i*,*r*_ ∈ ℂ are the hydrogen values attached to this carbon atom, and *d* represents the number of hydrogen atoms in the molecule.

(1) $$\begin{array}{c} \varphi \left(x\right)=v_{\text{C}}\overset{k}{\underset{r=1}{\sum }}\overset{d\leq 4}{\underset{i=1}{\prod }}\left(x_{r}-h_{i,r}\right). \end{array}$$

If two or more molecules have less number of hydrogen than allowed, i.e., *d* < 4, then they are able to join together forming chains of molecules. These chains are namely hydrocarbon compounds. Throughout this work, compounds are made of *n* molecules: a linear chain of (*n* − 2)CH_2_ molecules with two CH_3_ molecules, one at each side of the CH_2_-chain [45]. In addition, a piecewise function *ψ* denoted as Equation (2) is associated to the compound representing its behavior due to an input *x*, where *L*_*t*_={*L*_*t*,1_,…, *L*_*t*,*k*_} for all *t*=0,…, *n* are bounds where molecules can act over the input space [45].

(2) $$\begin{array}{c} \psi \left(x\right)=\left\{\begin{array}{cc} \varphi_{1}\left(x\right), & L_{0,r}\leq x_{r}<L_{1,r},\\ \cdots & \cdots \\ \varphi_{n}\left(x\right), & L_{n-1,r}\leq x_{r}\leq L_{n,r}. \end{array}\right. \end{array}$$

Lastly, different compounds can be selected and added up to form complex structures called mixtures. In AHN, a mixture is a linear combination of behavior compounds *ψ*_*j*_ in finite ratios *α*_*j*_, representing the weights of compounds, as expressed in the following equation:

(3) $$\begin{array}{c} S\left(x\right)=\overset{c}{\underset{j=1}{\sum }}\alpha_{j}\psi_{j}\left(x\right). \end{array}$$

To this end, AHN is trained using the so-called AHN algorithm reported with detail in the literature [44–47].

#### 2.4.2. Fuzzy-Molecular Inference System

**Figure 1:**
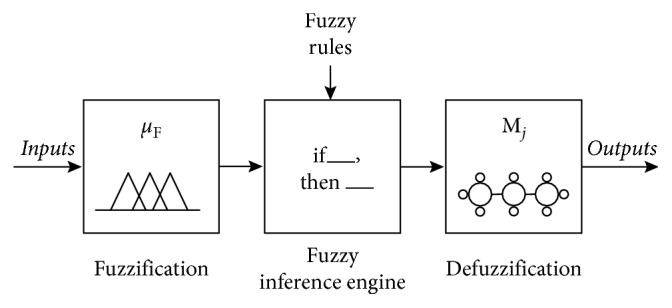
Block diagram of the fuzzy-molecular inference system.

As mentioned above, FMI is an ensemble of fuzzy logic and AHN [43].  Figure 1 shows the block diagram of the FMI. It consists of three main steps: fuzzification, fuzzy inference engine, and defuzzification based on AHN.

Fuzzification and fuzzy inference engine steps are quite similar to fuzzy logic. An input *x* is mapped to a set of fuzzy sets, using membership functions. Then, an inference operation, represented as a fuzzy rule, is applied to obtain a consequent value *y*_*p*_. Considering the *p*th fuzzy rule *R*_*p*_ denoted as Equation (4), inference computes *y*_*p*_ in terms of an artificial hydrocarbon compound with *n* molecules, *M*_*j*_, each one with function compound *φ*_*j*_(*x*) for all *j*=1,…, *n*. In this work, the membership value of *y*_*p*_ is calculated using the min function, expressed as *μ*_Δ_(*x*_1_,…, *x*_*k*_), over the fuzzy inputs.

(4) $$\begin{array}{c} R_{p}\text{:}\;\;\mathrm{if}\;\;x_{1}\in A_{1}\;\wedge \;\cdots \;\wedge \;x_{k}\in A_{k},\\ \mathrm{then}\;\;y_{p}=\varphi_{j}\left(\mu_{\Delta }\left(x_{1},\ldots ,x_{k}\right)\right). \end{array}$$

In the defuzzification step, it computes the crisp output value *y*, using the center of gravity approach [43], as expressed in the following equation:

(5) $$\begin{array}{c} y=\frac{\sum_{p}\mu_{\Delta }\left(x_{1},\ldots ,x_{k}\right)\cdot y_{p}}{\sum_{p}\mu_{\Delta }\left(x_{1},\ldots ,x_{k}\right)}. \end{array}$$

### 2.5. Nature-Inspired Control System for Mobile Robot Navigation

**Figure 2:**
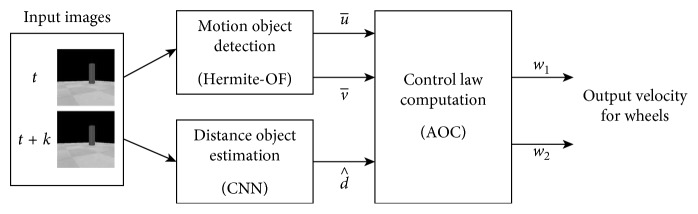
Block diagram of the nature-inspired control system.

In this work, we propose a nature-inspired controller system for mobile robot navigation implementing an AOC enhanced with Hermite OF and CNN. Particularly, this approach requires only a single camera mounted in the robot, implementing in this way an egocentric vision system. No other sensors are required for this controller.  Figure 2 shows the block diagram of the proposed control system. It consists of the following steps: (i) motion object detection, (ii) distance object estimation, and (iii) control law computation.

#### 2.5.1. Motion Object Detection

The motion object detection step considers determining the relative displacement that an object located in front of the robot is performing. This information is very useful when dealing with mobile obstacles. To compute the estimated relative displacement of the object in an image, the Hermite OF method is employed in a similar way as proposed in [21].

First, two adjacent gray images, *I*_*t*_ in time *t* and *I*_*t*+*k*_ in time *t*+*k*, are acquired by the single camera. Then, the Hermite OF algorithm computes the relative displacements of objects between these images. This procedure outputs a map of displacements. These relative displacements are decomposed in both horizontal (*u*) and vertical (*v*) components. Assuming that the mobile obstacle presents more displacement than the rest of the scene, then a mean value per axis, $\bar{u}$ and $\bar{v}$, is calculated for estimating the relative displacement of the object. To this end, the mean values $\bar{u}$ and $\bar{v}$ are passing as inputs to the AOC explained below.

#### 2.5.2. Distance Object Estimation

The distance object estimation step considers determining the distance of the robot from an object using the single camera. However, computing this value in an image is a challenging task [4]. In this way, we propose to use a CNN for estimating the distance of an object in the environment.

To do so, the same two images *I*_*t*_ and *I*_*t*+*k*_ are occupied in this step. Once again, the Hermite OF method is computed using these images. The output map of displacements obtained with this method is converted to two images *I*_*u*_ and *I*_*v*_ related to the horizontal and vertical displacements, respectively. Considering that the size of the original images *I*_*t*_ and *I*_*t*+*k*_ is *m* × *n*, we use *I*_*u*_ and *I*_*v*_ to form a new image *I*_*uv*_=[*I*_*u*_, *I*_*v*_] of size *m* × 2*n*. Then, the latter image is used as the input of the CNN.

**Figure 3:**
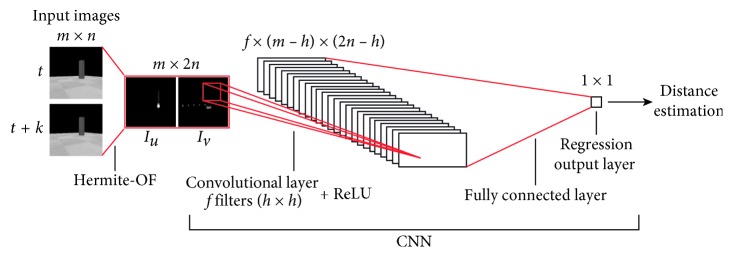
CNN topology of the proposed distance object estimation approach.

The CNN has a convolutional layer with *f* filters of size *h* × *h* that is used for calculating the feature representations of the input image. It is followed by a rectified linear unit (ReLU) layer, and finally it has a fully connected layer, with output size 1, together with a regression layer to perform a high-level reasoning for estimating the distance $\hat{d}$ to an object. To implement the CNN, we previously obtained the features of motion leaving to the CNN only the reasoning process from the spatial localization of motion to the distance estimation. In this case, the proposed architecture could be minimal, and the size of the training data could also be small enough to learn robust features.  Figure 3 shows the topology of the proposed CNN in the distance object estimation step. For training purposes, we prepared a dataset using ultrasonic sensors as target values, as explained later on. To this end, the distance estimation $\hat{d}$ is passed as input to the AOC explained below. No other architectures were tried for this work.

#### 2.5.3. Control Law Computation

**Figure 4:**
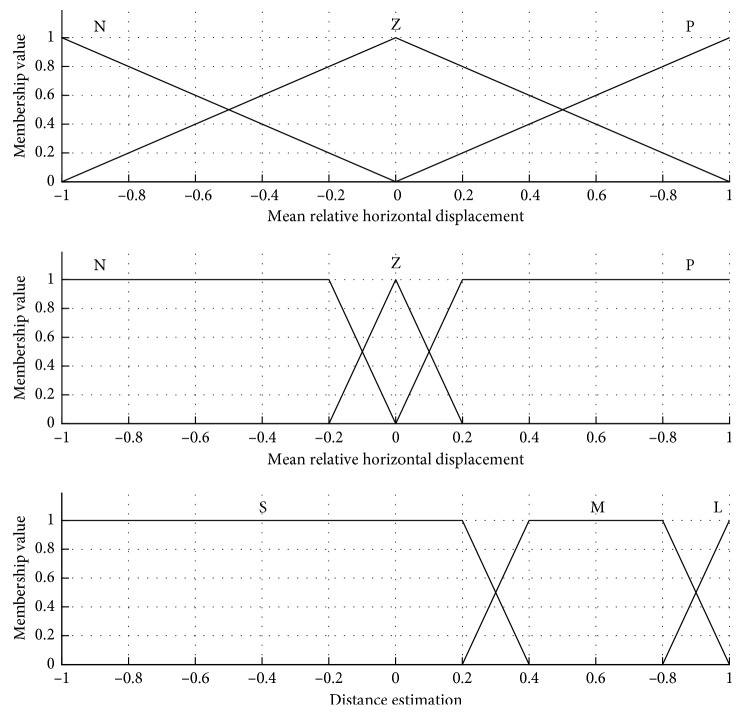
Input membership functions in the AOC: (a) mean relative horizontal displacement, (b) mean relative vertical displacement, and (c) distance estimation.

The last step of the nature-inspired control system is the control law computation using the AOC. As shown in Figure 2, three inputs are defined as follows: the mean values $\bar{u}$ and $\bar{v}$, representing the relative displacement components of a mobile object, are partitioned in three fuzzy sets like “negative” (N), “zero” (Z), and “positive” (P), while the distance estimation $\hat{d}$, from the robot to an object, is partitioned also in three fuzzy sets like “small” (S), “medium” (M), and “large” (L). Particularly for this work, the proposed input membership functions are depicted in Figure 4.

**Figure 5:**
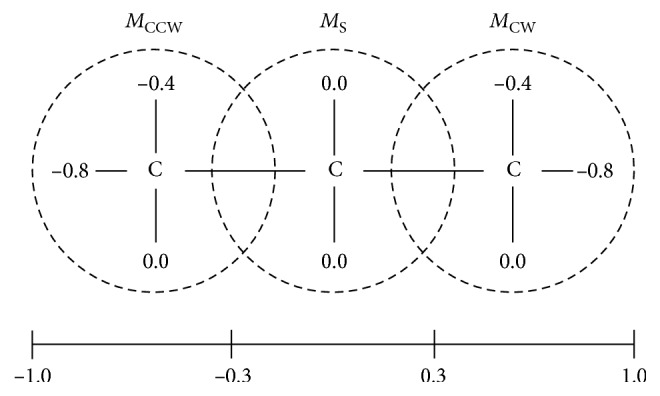
Output molecular partitions in the AOC for the velocity calculation in both wheels *w*_1_ and *w*_2_.

In addition, Table 1 presents the set of fuzzy rules designed for the mobile robot navigation task. These rules consider obstacle avoidance and free navigation of the robot. To this end, Figure 5 shows the artificial hydrocarbon compound developed for this work. It comprises three molecules representing the output velocity of the wheels *w*_1_ and *w*_2_ in the robot like “counterclockwise” (CCW), “stop” (S), and “clockwise” (CW).

## 3. Results and Discussion

In order to validate our proposed nature-inspired controller system for mobile robot navigation implementing an AOC enhanced with Hermite OF and CNN, we develop a set of experiments to independently prove each of the components of the system in an incremental fashion. These experiments measure the output response of (i) avoiding a mobile obstacle using the Hermite OF, (ii) avoiding a mobile obstacle as well as free navigating using the Hermite OF and AOC, (iii) avoiding a fixed obstacle using CNN, and (iv) avoiding fixed and mobile obstacles as well as free navigating using the whole proposed nature-inspired controller system.

In this work, the performance of the robot navigation is evaluated objectively by computing some metrics related to the security and smoothness of the control navigation response. Three security indexes are used to evaluate the distance between the robot trajectory and the location of obstacles [48]:

- SM1: it measures the mean distance between the trajectory of the robot to the closest obstacle.
- SM2: it measures the minimum distance between the trajectory of the robot and the mean distance to all obstacles.
- SM3: it measures the minimum distance over the trajectory of the robot to the closest obstacle.

For the security metrics, larger values of the indexes represent a better behavior in the robot navigation, since they intuitively measure the security distance at which the robot is located away from the obstacles.

In addition, three smoothness indexes are employed to indirectly evaluate the consistency between the decision-action relationship of the control navigation in the robot and the ability to react to events with sufficient speed [48]. The bending energy (*B*_E_) measures the energy for steering or bending during the trajectory, and it is calculated as Equation (6), where *k*_*t*_ represents the curvature of the trajectory *f*(*t*) computed as Equation (7), *n* is the number of points in the discrete trajectory, and *t* is the current time.

(6) $$\begin{array}{c} B_{\text{E}}=\frac{1}{n}\overset{n}{\underset{t=1}{\sum }}k_{t}^{2}, \end{array}$$

(7) $$\begin{array}{c} k_{t}=\frac{f^{\prime \prime }\left(t\right)}{{\left(1+f^{\prime }{\left(t\right)}^{2}\right)}^{3/2}}. \end{array}$$

The smoothness metric that considers bending energy over time (TB_*E*_) is calculated as follows:

(8) $$\begin{array}{c} {\text{TB}}_{\text{E}}=\overset{n}{\underset{t=1}{\sum }}k_{t}^{2}. \end{array}$$

Lastly, the smoothness of curvature (*S*_*k*_) measures the change in curvature *k* all along the trajectory *f* with length *L* performed by the robot navigation over time, and it can be expressed as follows:

(9) $$\begin{array}{c} S_{k}=\frac{\int_{0}^{L}{\left(dk/dt\right)}^{2}ds}{t}. \end{array}$$

For smoothness metrics, smaller values close to zero represent smooth curvatures in trajectory and less energy in the performance.

### 3.1. Mobile Obstacle Avoidance Using Real-Time Hermite OF

**Figure 6:**
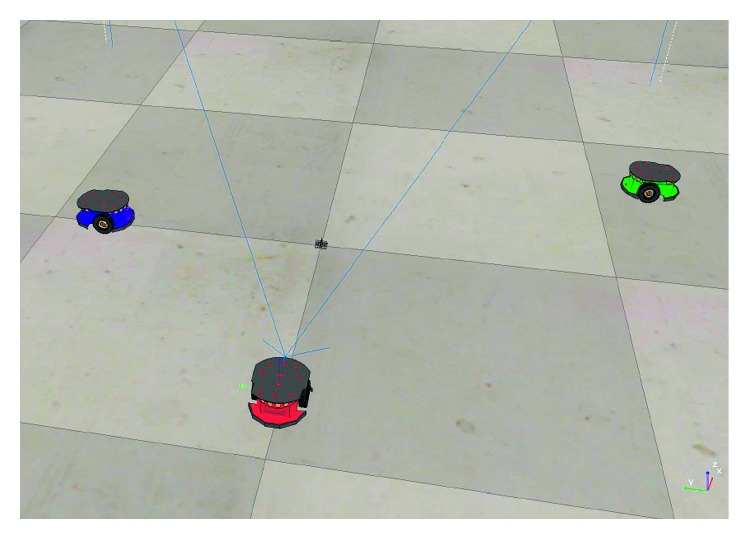
Scenario developed for testing avoidance of mobile obstacles (green and blue robots).

The first experiment aims to measure the output response of avoiding mobile obstacles using the real-time Hermite OF.  Figure 6 shows the initial configuration of the environment. It considers two mobile robots as obstacles (green and blue). The red robot shown in the scene is the one with the RT-HOF. For implementation purposes, this method is based on the work of [20].

In a nutshell, two consecutive images are acquired, and the Hermite OF is computed getting the relative displacements decomposed in horizontal *u* and vertical *v* components. Then, the mean values of the components, $\bar{u}$ and $\bar{v}$, are calculated. In addition, the relative direction *θ* between vectors *u* and *v* are computed such that the mean angle $\bar{\theta }$ is obtained. Lastly, a simple set of rules are considered for avoiding mobile obstacles, as shown in Algorithm 1. In this work, the threshold values in the set of rules were set experimentally: *T*_*u*_=0.05, *T*_*v*_=−0.2, *T*_*l*_=40, *T*_*u*_=130, *T*_*m*_1__=90, and *T*_*m*_2__=130.

**Figure 7:**
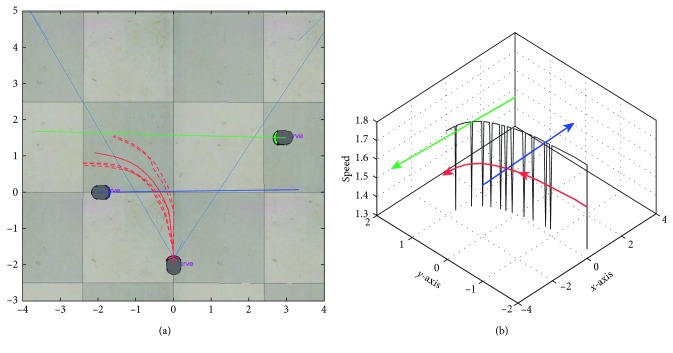
Output response using the real-time Hermite OF method. (a) Trajectory of the target robot: five attempts (dashed lines) and mean (red line); (b) speed representation of the target robot (dark line) over the trajectory.

**Figure 8:**
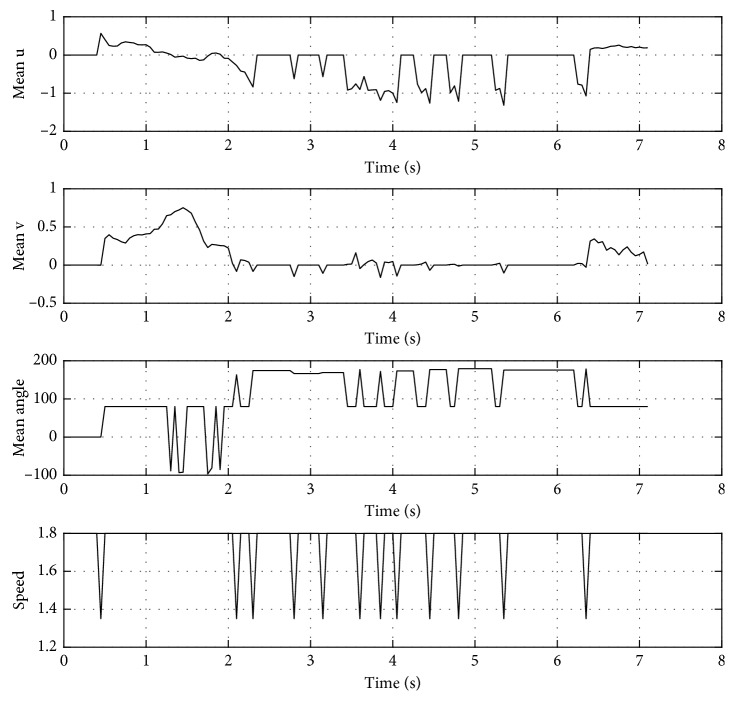
Inputs ($\bar{u},\;\;\bar{v},\;\;\bar{\theta }$) and output (*speed*) values for the controller.

In Figure 7, it is shown the output trajectories when using the RT-HOF method. Five attempts were run (reported as the dashed red lines), and the mean trajectory (strong-red line) is depicted in Figure 7(a), while Figure 7(b) shows the speed of the target robot over its trajectory. Notice that the red robot steers to the left trying to avoid the blue robot that is going to the right. In this particular case, the avoidance procedure controller (Algorithm 1) was designed for avoiding obstacles in the backwards. In addition, the trajectory of the target robot when dealing with the green robot is done by positioning in parallel to the direction of it. From Figure 7(b), it is observed that the red robot decreases its velocity once it detects another mobile object. Also, the velocity is discrete since the controller is based on a set of crisp rules. Moreover, Figure 8 shows the inputs ($\bar{u},\;\;\bar{v},\;\;\bar{\theta }$) and output (*speed*) values of the controller, where speed is related to the linear velocity of the target robot. It can be observed that $\bar{u}$ has more influence than $\bar{v}$ when objects are close to the robot. In addition, $\bar{\theta }$ is correlated to the steering action of the robot.

In addition, security and smoothness indexes are summarized in Table 2 for each of the attempts and the mean performance. As shown in the indexes, security values consider that the trajectories obtained allow the robot to be far from the mobile obstacles (mean minimum distance reached of 0.63 m). Smoothness indexes are close to zero meaning that the trajectories of the robot do not represent abrupt changes.

To this end, in this experiment, the target robot did not collide with any of the mobile obstacles in all the attempts.

### 3.2. Mobile Obstacle Avoidance and Free Navigation Using AOC

Using the AOC with real-time Hermite OF and the inferred distance, from the CNN training step, as inputs, we tested the wheeled robot such that it can navigate freely in an environment avoiding obstacles.

**Figure 9:**
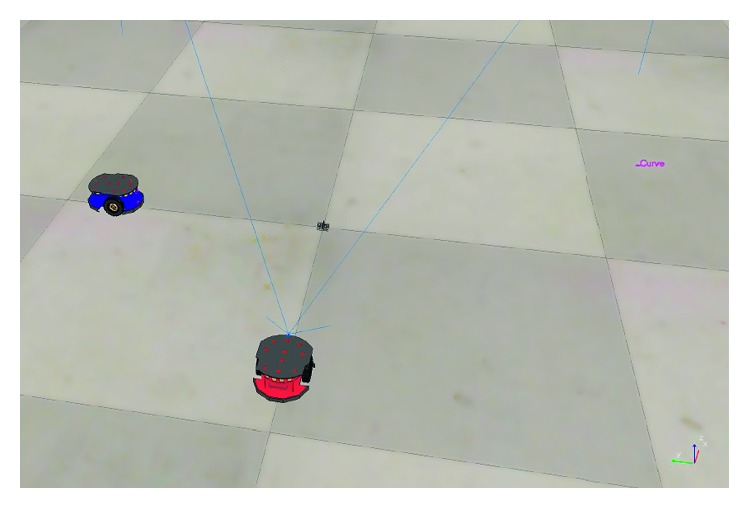
Scenario developed for testing avoidance of a mobile obstacle (blue robot).

Figure 9 shows the initial configuration of the environment. It considers one mobile robot as obstacle (blue). The red robot shown in the scene is the one with the RT-HOF method and the AOC. For implementation purposes, this method is based on the work of [21].

**Figure 10:**
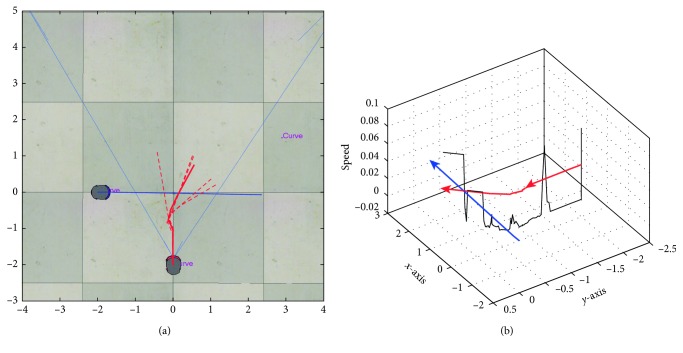
Output response using RT-HOF and AOC. (a) Trajectory of the target robot: five attempts (dashed lines) and mean (red line); (b) speed representation of the target robot (dark line) over the trajectory.

**Figure 11:**
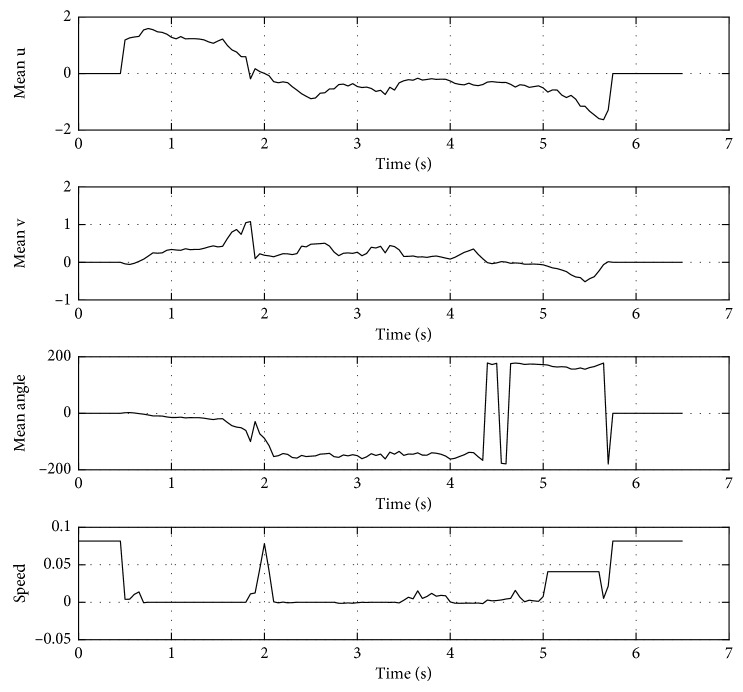
Inputs ($\bar{u},\;\;\bar{v},\;\;\bar{\theta }$) and output (*speed*) values for the controller.

The output trajectories when using RT-HOF and AOC are shown in Figure 10. Five attempts were run (reported as the dashed red lines), and the mean trajectory (strong red line) is also depicted in Figure 10(a). Again, Figure 10(b) shows the velocity of the target robot over its trajectory. In this experiment, the target robot outputs a trajectory that prevents collision with the mobile obstacle (running from left-to-right) by reducing the velocity and steering a little bit to the left. Then, it corrects its trajectory, but different decisions are taken. It mainly happens due to the slight variations in the position of the mobile obstacle. Moreover, the velocity appreciated in Figure 10(b) is smoother than in the previous experiment. It is important for robotics implementations because it better regulates electrical current in the actuators.  Figure 11 shows the inputs ($\bar{u},\;\;\bar{v},\;\;\bar{\theta }$) and output (*speed*) values for this controller.

In terms of the security and smoothness indexes, Table 3 summarizes the results for each of the attempts and the mean performance. As shown in the indexes, security values consider that the trajectories obtained allow the robot to be far from the mobile obstacles (mean minimum distance reached of 0.78 m). Smoothness indexes prove minimal abrupt changes in the trajectories.

Again, in this experiment, the target robot did not collide with the mobile obstacle in all the attempts.

### 3.3. Fixed Obstacle Avoidance Using CNN

This experiment includes the proposed CNN with OF distance-object estimation model for an egocentric vision-based robot. The CNN was trained from scratch using the data (*𝒟*_cnn+of_) collected from a set of 10 attempts, with 860 samples each, between the robot and one fixed object. The dataset is balanced in terms of short and large distances. The dataset was divided into 70% training and 30% testing, randomly chosen. Then, images *I*_*u*_ and *I*_*v*_ representing the horizontal and vertical components of the OF were resized to 28 × 28, and then concatenating them to produce a single 28 × 56 image, *I*_*uv*_=[*I*_*u*_, *I*_*v*_].

To this end, the CNN with OF was trained by using the pairs {*I*_*uv*_, *d*}_*i*_ in the training set, where *d* is the sensor-based distance measure, at the *i*-element of the set. Lastly, the stochastic gradient descent method was used for training with initial learning rate 0.01, momentum 0.90, and mini-batch size of 16. *L*_2_ regularization was performed with *λ*=0.0001 term.

After that, the root mean square error (RMSE) from Equation (10) was employed for measuring the performance of the CNN over the remaining 30% of the dataset, where *d*_*q*_ is the target distance measured from the sensor, ${\hat{d}}_{q}$ is the estimated distance from the CNN, and *N* is the size of the testing data. Also, the accuracy was measured as the number of times the difference between the target and estimated distances is below or equal to a threshold *ϵ*, as shown in Equation (11). After testing, the performance of the CNN with OF obtained 0.0591 in RMSE (meters) and 90.7% in accuracy with threshold *ϵ*=0.10 m.

(10) $$\begin{array}{c} \text{RMSE}=\sqrt{\frac{\sum_{q=1}^{N}{\left(d_{q}-{\hat{d}}_{q}\right)}^{2}}{N}}, \end{array}$$

(11) $$\begin{array}{c} \text{accuracy}=\frac{\sum_{q=1}^{N}\left\{\left|d_{q}-{\hat{d}}_{q}\right|\leq \epsilon \right\}}{N}. \end{array}$$

**Figure 12:**
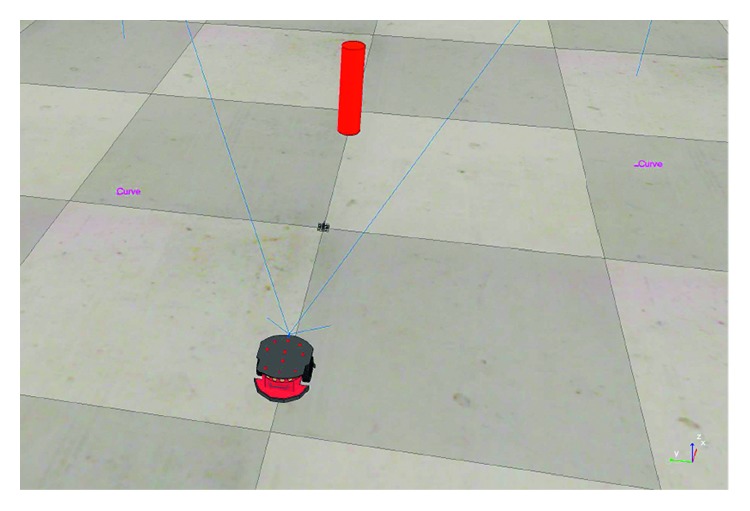
Scenario developed for testing avoidance of a fixed obstacle (cylinder).

Thus, in Figure 12, it is depicted the initial configuration of the environment. It considers one fixed obstacle (red cylinder). The red robot shown in the scene is the one with the CNN-based distance estimation model that is used during the whole time in the experiment.

**Figure 13:**
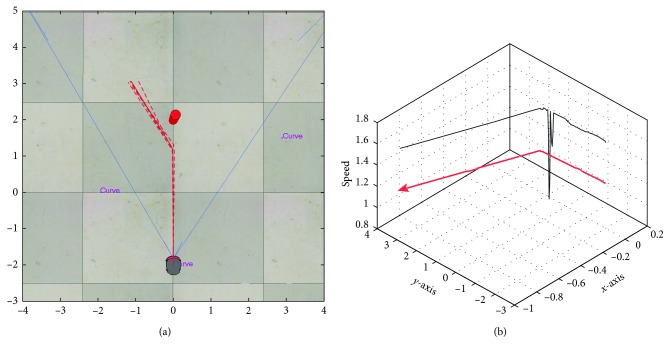
Output response using the CNN-based distance estimation method. (a) Trajectory of the target robot: five attempts (dashed lines) and mean (red line); (b) speed representation of the target robot (dark line) over the trajectory.

**Figure 14:**
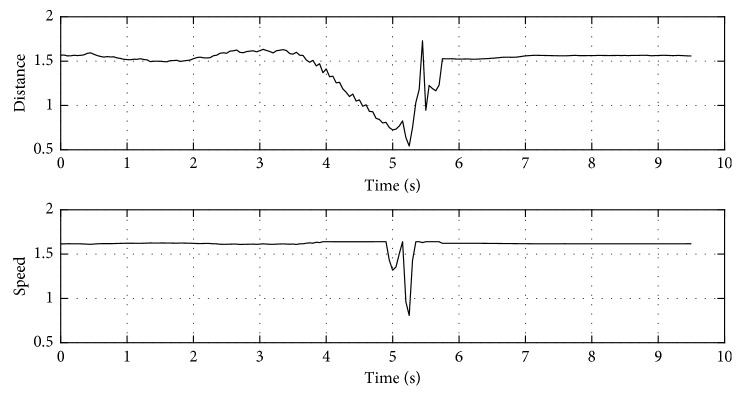
Input ($\hat{d}$) and output (*speed*) values for the controller.

The output trajectories when using the CNN-based distance estimation model are shown in Figure 13. Five attempts were run (reported as the dashed red lines), and the mean trajectory (strong red line) is also depicted (Figure 13(a)). It also shows the velocity of the target robot over its trajectory (Figure 13(b)). As shown, the target robot goes forward until it detects the proximity of the obstacle by estimating the distance to it with the CNN model. Also, an implemented AOC determines the velocities of the wheels, aiming the robot steering to the left.  Figure 14 reports the input ($\hat{d}$) and output (*speed*) values for the controller. The behavior of the red robot is very precise, and during this experiment it did not collide with the cylinder.

In terms of the security and smoothness indexes, Table 4 summarizes the results for each of the attempts and the mean performance. As shown in the indexes, the robot reaches close positions to the obstacle, with a minimum distance of 0.42 m securing minimal risks in the robot. Smoothness indexes prove minimal abrupt changes in the trajectories when dealing with a fixed obstacle.

### 3.4. Obstacle Avoidance and Free Navigation Using the Proposed Controller

**Figure 15:**
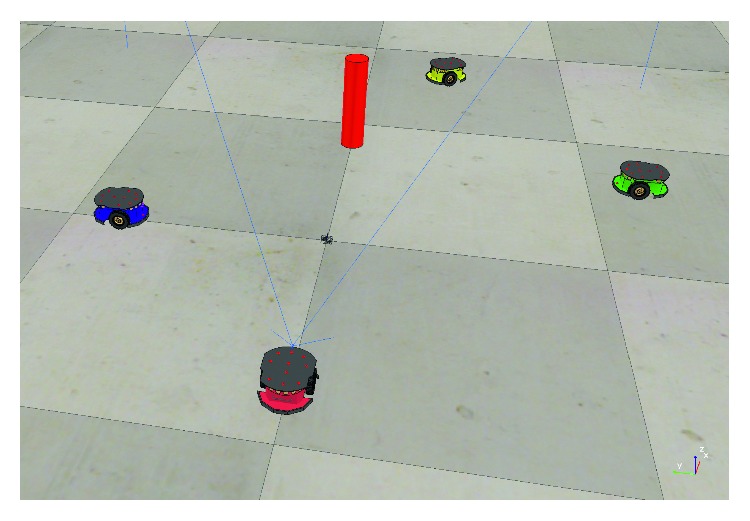
Scenario developed for testing obstacle avoidance and free navigation.

The last experiment aims to measure the output performance of the proposed nature-inspired control system for avoiding fixed and mobile obstacles and free navigating over the environment.  Figure 15 shows the test scenario for this experiment. It comprises one fixed obstacle (red cylinder) and three mobile obstacles (green, blue, and yellow robots), as well as the target robot (red robot). The scene shown in Figure 15 represents the initial conditions. The target robot is the one with the proposed controller implemented as described in Section 2.5.

**Figure 16:**
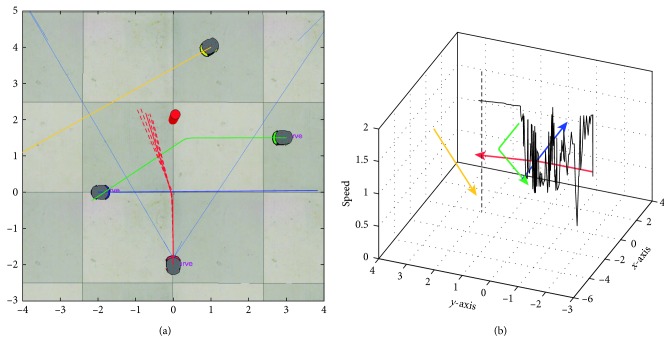
Output response using the proposed nature-inspired control system. (a) Trajectory of the target robot: five attempts (dashed lines) and mean (red line); (b) speed representation of the target robot (dark line) over the trajectory.

Figure 16 shows the output trajectories when using the proposed controller. Five attempts were run (reported in dashed red lines), and the mean trajectory (strong red line) is depicted in Figure 16(a), while Figure 16(b) shows the speed of the target robot over its trajectory. As noticed, the target robot dealt with both fixed and mobile obstacles. For instance, the first object seen by the robot is the cylinder. However, the blue robot is then crossed in front of the target robot. In this situation, the red robot decreases its velocity and steers to the left slightly. Once the blue robot is out of range, the target robot moves forward decreasing its velocity again while the green robot is moving around. Later on, the green robot is out of the vision of the red robot, but the red cylinder is relatively closer. In that way, the target robot steers again to the left to avoid collision with the cylinder. At last, the yellow robot is not seen by the target robot, so it goes straight.

**Figure 17:**
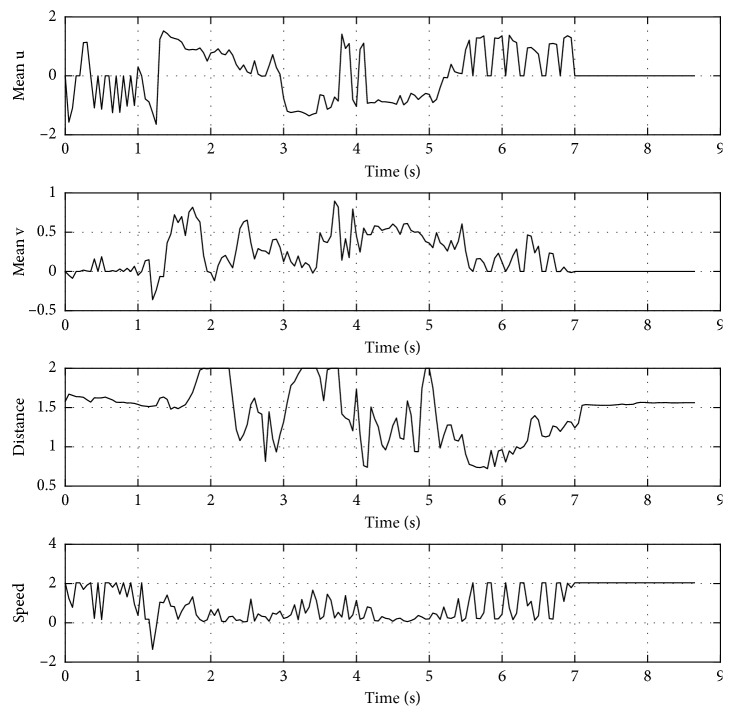
Inputs ($\bar{u},\;\;\bar{v},\;\;\hat{d}$) and output (*speed*) values for the proposed controller.

In addition, Figure 17 shows the inputs ($\bar{u},\;\;\bar{v},\;\;\hat{d}$) and output (*speed*) values of the proposed controller. Notice that the combined behavior of previous experiments is obtained in this proposal. For instance, the speed of the target robot decreases when $\bar{u}$ is positive and $\bar{v}$ is about zero (e.g., detecting a mobile obstacle), or when $\hat{d}$ is small (e.g., detecting a fixed obstacle). The combination of $\bar{u}$ and $\bar{v}$ is correlated to the steering of the target robot.

We measure the security and smoothness of the trajectories, as shown in Table 5. It summarizes the results for each of the attempts and the mean performance. From these metrics, it can be observed that the robot maintained the minimal secure distance of 0.61 m from obstacles, reducing the risks for collisions. In terms of smoothness, this control system allowed the robot to navigate without larger changes in curvature.

It is remarkable to say that the vision of the robot might have more than one object at the time, increasing the difficulty for determining the best action of the robot. To this end, the target robot did not collide in any of the attempts conducted in this experiment, validating that the proposed controller can be implemented for avoiding obstacles and free navigating in scenarios where there are both fixed and mobile obstacles.

### 3.5. Discussion

In this work, we estimate the distance to the objects instead of a depth map of the scene as reported in several studies in the literature. For this task, we use a monocular camera to infer the distance and the object motion in contrast to the applications using stereo vision systems that are highly computational resource consuming. An advantage of our proposal is the use of two kinds of measures (motion and distance) that allow avoiding fixed and mobile obstacles. In addition, our method needs less data for training the distance estimator and no training data to compute the OF field, in contrast with the OF-based CNN approaches.

The use of these cognitive strategies complement the robot control, in contrast with traditional applications in robotic vision. Hence, it is the first time that CNN training was carried out using ultrasound sensors. In addition, the AOC is implemented for handling uncertain information such as the estimations from the inputs, giving robustness to the control system. The control strategy uses the OF features directly without need of path planning over depth maps.

Some drawbacks of the proposed approach are the following: (1) this approach does not consider multiple objects in the scene, so complementary treatment would be required. (2) In the experiments, we use the robot simulator V-REP that recreates the conditions of the physical world quite accurately, but further investigation in real conditions are necessary.

## 4. Conclusions

In this paper, we presented an autonomous navigation system to be applied in RAL. We propose an integrated system including a vision sensor, real-time Hermite OF, and distance estimation by CNN to introduce to the intelligent robot controller based on AOC. We use the OF for motion estimation and CNN for distance inference to the objects, making this application suitable for avoiding fixed and mobile targets. Particularly, we use a monocular camera for the whole task.

We did four experiments to test different scenarios: using only OF and on-off control for mobile obstacles, a combination of OF and AOC, a CNN-based distance estimation and AOC for fixed obstacles, and the integration of OF, CNN-based distance estimation, and AOC. Simulation results were done in V-REP software and the results confirm that our approach is useful for obstacle avoidance and free navigation. In all test scenarios, there was never a collision with the objects using our proposal. A quantitative analysis was done using the security and smoothness metrics applied to the control navigation response. These quantitative metrics suggest that using the AOC strategy allows to avoid obstacles in a comfortable way and with minimal abrupt changes in the trajectory. To this end, the experiments confirm that a monocular camera can be applied for robot navigation tasks.

The proposed approach successfully combines a bioinspired OF method, a CNN technique for distance inference, and a novel hybrid fuzzy logic and artificial hydrocarbon networks controller system. This integration loosely simulates a high cognitive vision strategy that allows analyzing holistic information from the egocentric point of view of the mobile robot.

As future work, we will test our approach over RAL in real scenarios in order to improve the navigation performance of mobile robots in presence of dynamic environments typically found in the context of elderly people places.

## Figures and Tables

**Algorithm 1:**
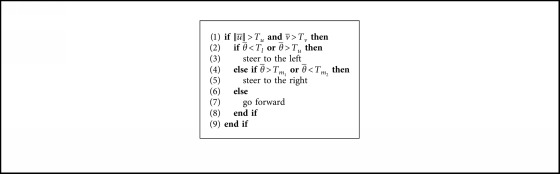
Set of rules for the first experiment used in obstacle avoidance.

**Table 1:** Fuzzy rules of the AOC for mobile robot navigation.

$\bar{u}$	$\bar{v}$	$\hat{d}$	*w*_1_	*w*_2_
*N*	*N*	*S*	*M*_CW_	*M*_CCW_
*N*	*N*	*M*	*M*_CW_	*M*_S_
*N*	*N*	*L*	*M*_CW_	*M*_S_
*N*	*Z*	*S*	*M*_CCW_	*M*_CW_
*N*	*Z*	*M*	*M*_S_	*M*_CW_
*N*	*Z*	*L*	*M*_CW_	*M*_CW_
*N*	*P*	*S*	*M*_CCW_	*M*_CW_
*N*	*P*	*M*	*M*_S_	*M*_CW_
*N*	*P*	*L*	*M*_S_	*M*_CW_
*Z*	*N*	*S*	*M*_CW_	*M*_CCW_
*Z*	*N*	*M*	*M*_CW_	*M*_S_
*Z*	*N*	*L*	*M*_CW_	*M*_S_
*Z*	*Z*	*S*	*M*_CCW_	*M*_CW_
*Z*	*Z*	*M*	*M*_S_	*M*_CW_
*Z*	*Z*	*L*	*M*_CW_	*M*_CW_
*Z*	*P*	*S*	*M*_CCW_	*M*_CW_
*Z*	*P*	*M*	*M*_S_	*M*_CW_
*Z*	*P*	*L*	*M*_S_	*M*_CW_
*P*	*N*	*S*	*M*_CW_	*M*_CCW_
*P*	*N*	*M*	*M*_CW_	*M*_S_
*P*	*N*	*L*	*M*_CW_	*M*_S_
*P*	*Z*	*S*	*M*_CCW_	*M*_CW_
*P*	*Z*	*M*	*M*_S_	*M*_CW_
*P*	*Z*	*L*	*M*_CW_	*M*_CW_
*P*	*P*	*S*	*M*_CCW_	*M*_CW_
*P*	*P*	*M*	*M*_S_	*M*_CW_
*P*	*P*	*L*	*M*_S_	*M*_CW_

**Table 2:** Security and smoothness indexes for the robot navigation using the real-time Hermite OF.

Attempt	SM1 (m)	SM2 (m)	SM3 (m)	TB_E_	*B*_E_	*S*_*k*_
1	1.4796	1.3166	0.62518	4.6097*e*−05	7.8663*e*−08	1.0344*e*−06
2	1.4756	1.415	0.7146	4.518*e*−05	7.4678*e*−08	1.0005*e*−06
3	1.6943	1.2665	0.58068	4.9117*e*−05	7.5448*e*−08	9.6104*e*−07
4	1.7563	1.2261	0.54734	4.6815*e*−05	6.5844*e*−08	9.2914*e*−07
5	1.6145	1.364	0.66258	4.471*e*−05	7.5017*e*−08	9.8786*e*−07
Mean	1.6041	1.3176	0.62608	4.6384*e*−05	7.393*e*−08	9.8259*e*−07

**Table 3:** Security and smoothness indexes for the robot navigation using the RT-HOF and AOC.

Attempt	SM1 (m)	SM2 (m)	SM3 (m)	TB_E_	*B*_E_	*S*_*k*_
1	0.7473	1.6509	0.7473	9.7704*e*−05	1.84*e*−07	2.4774*e*−06
2	0.74157	1.6427	0.74157	7.2661*e*−05	1.5044*e*−07	1.4464*e*−06
3	0.85645	1.5416	0.85645	0.00013544	2.7307*e*−07	3.3304*e*−06
4	0.76235	1.9285	0.76235	7.6021*e*−05	1.5483*e*−07	1.8303*e*−06
5	0.79618	1.6155	0.79618	0.00010674	2.3408*e*−07	2.4665*e*−06
Mean	0.78077	1.6758	0.78077	9.7714*e*−05	1.9928*e*−07	2.3102*e*−06

**Table 4:** Security and smoothness indexes for the robot navigation using the CNN-based estimation method.

Attempt	SM1 (m)	SM2 (m)	SM3 (m)	TB_E_	*B*_E_	*S*_*k*_
1	0.4241	1.9076	0.4241	3.5541*e*−05	4.58*e*−08	5.5335*e*−07
2	0.46384	1.9298	0.46384	3.2683*e*−05	4.3577*e*−08	5.6792*e*−07
3	0.37958	1.9096	0.37958	3.7597*e*−05	4.9339*e*−08	6.4338*e*−07
4	0.44578	1.9168	0.44578	3.4012*e*−05	4.3162*e*−08	5.6113*e*−07
5	0.37746	1.909	0.37746	3.0383*e*−05	3.8754*e*−08	5.2752*e*−07
Mean	0.41815	1.9146	0.41815	3.4043*e*−05	4.4127*e*−08	5.7066*e*−07

**Table 5:** Security and smoothness indexes for the robot navigation using the proposed nature-inspired control system.

Attempt	SM1 (m)	SM2 (m)	SM3 (m)	TB_E_	*B*_E_	*S*_k_
1	1.1638	1.9244	0.6004	0.00079652	7.3548*e*−07	1.2218*e*−05
2	1.2589	2.0064	0.66768	0.00071704	6.3455*e*−07	9.8604*e*−06
3	1.1763	1.9168	0.56143	0.00089058	7.6973*e*−07	1.1408*e*−05
4	1.2612	1.9481	0.71119	0.00060311	5.3945*e*−07	9.062*e*−06
5	1.1768	1.9034	0.53153	0.00085002	7.7275*e*−07	1.2863*e*−05
Mean	1.2074	1.9398	0.61445	0.00077145	6.9039*e*−07	1.1082*e*−05

## Data Availability

The dataset used in this work was collected by the authors, and it can be found in http://sites.google.com/up.edu.mx/robotflow/.
